# Evaluating the Outcomes of Managing Displaced Clavicular Fractures by Using Precontoured Clavicular Plates

**DOI:** 10.7759/cureus.66095

**Published:** 2024-08-03

**Authors:** Krishna M, Shagnik Paul, Rakesh K Gupta, Amandeep Mittal, Sanju Bishnoi, Aksha M Garg, Manmeet Malik, Abhay Choudhary, Gaurav K Agrawal

**Affiliations:** 1 Orthopaedics, Pandit Bhagwat Dayal Sharma Post Graduate Institute of Medical Sciences, Rohtak, IND; 2 Orthopaedics, All India Institute of Medical Sciences, New Delhi, New Delhi, IND

**Keywords:** complication, functional and radiological outcome, constant-murley score, locking compression plating, clavicle fractures

## Abstract

Background and objective

Midshaft clavicular fractures were managed conservatively in the past, with a significant incidence of nonunion and poor functional outcomes in displaced fractures. Anatomically precontoured clavicle plates, since their introduction, have proved to be a superior method for managing these fractures. While open reduction and internal fixation of displaced clavicular fractures with plates have produced successful functional outcomes, complications like plate prominence, scar, postoperative numbness, wound dehiscence, refracture, and infection continue to discourage surgeons from plating these fractures. This study aimed to evaluate whether the precontoured 3.5-mm locking compression plate (LCP) for the clavicle is effective in the management of displaced clavicular fractures with minimum risk of complications.

Methods

A prospective observational study was conducted among 26 patients with displaced clavicular fractures that were managed with 3.5-mm precontoured LCP. The functional outcome was assessed by using the Constant-Murley Score (CMS) and healing was assessed radiographically six months postoperatively.

Results

Twenty-five patients were available for the final follow-up at the end of 24 weeks. All of them achieved excellent functional scores. The mean CMS was 94.9. No complication was observed in 85% of the cases. Implant failure was observed in both fractures of a bilateral clavicle fracture patient within a month of surgery. Implant irritation without prominence was seen in one patient and another had a prominent postoperative scar. The mean time for the radiological union was 13.8 weeks with union time ranging from three to five months.

Conclusions

Based on our findings, employing 3.5-mm precontoured clavicular LCPs is a useful technique that can provide good functional outcomes in displaced clavicular fractures.

## Introduction

Clavicular fractures account for around 2.5-10% of all fractures, reported as being the most common fracture in the body [1]. The mechanism of such injuries includes a fall on an outstretched hand or a direct blow to the shoulder. Most clavicular fractures (70-80%) occur in the midshaft where the typical compressive forces acting on the narrow cross-section of the bone combine and cause bony failure due to the lack of muscular and ligamentous attachments in this area [2]. The sternocleidomastoid pulls the medial segment superiorly while the weight of the arm pulls the lateral segment inferiorly through the coracoclavicular ligaments. Pectoralis major and latissimus dorsi also pull the lateral segment inferomedially leading to shortening.

Many authors have reported that anatomical reduction of the clavicle results in lower nonunion rates and better functional outcome scores [1,3,4,5]. However, the goal of clavicle fracture treatment is not only to achieve union but also to minimize dysfunction and improve cosmesis. Substantial comminution and segmental fracture patterns are believed to be contraindications for using intramedullary devices. Also, fixation with an intramedullary nail carries the risk of telescoping, secondary shortening, and nail migration, which may damage surrounding vital structures like the brachial plexus, subclavian vessels, and lungs. However, they offer the advantage of a smaller incision, minimal soft tissue disruption, and lesser hardware prominence [6].

Several studies still indicate that despite encouraging results with plate fixation of displaced clavicular fractures, the problem of complications in the form of plate prominence, scar, postoperative numbness, wound dehiscence, refracture, infection, and nonunion remains. It has been suggested in the literature that functional requirements, location, expectations of the patient, comminution, and nature of the fracture must be thoroughly assessed before surgery in terms of the risk-benefit ratio [7-8]. This study was therefore undertaken to evaluate whether the precontoured clavicular plate is effective in the management of displaced clavicular fractures with minimum risk of complications.

## Materials and methods

The study involved 26 patients (with one patient having a bilateral clavicle fracture) with 27 fractures.

Inclusion and exclusion criteria

All clavicular fractures with the following features were included in the present study: displacement >2 cm, shortening, comminution, segmental fracture, obvious clinical deformity, floating shoulder, fractures with skin tethering, fractures involving neurovascular compromise, old fractures with nonunion. Patients with pathological fractures, undisplaced fractures, and those aged <18 years were excluded.

Operative steps

**Figure 1:**
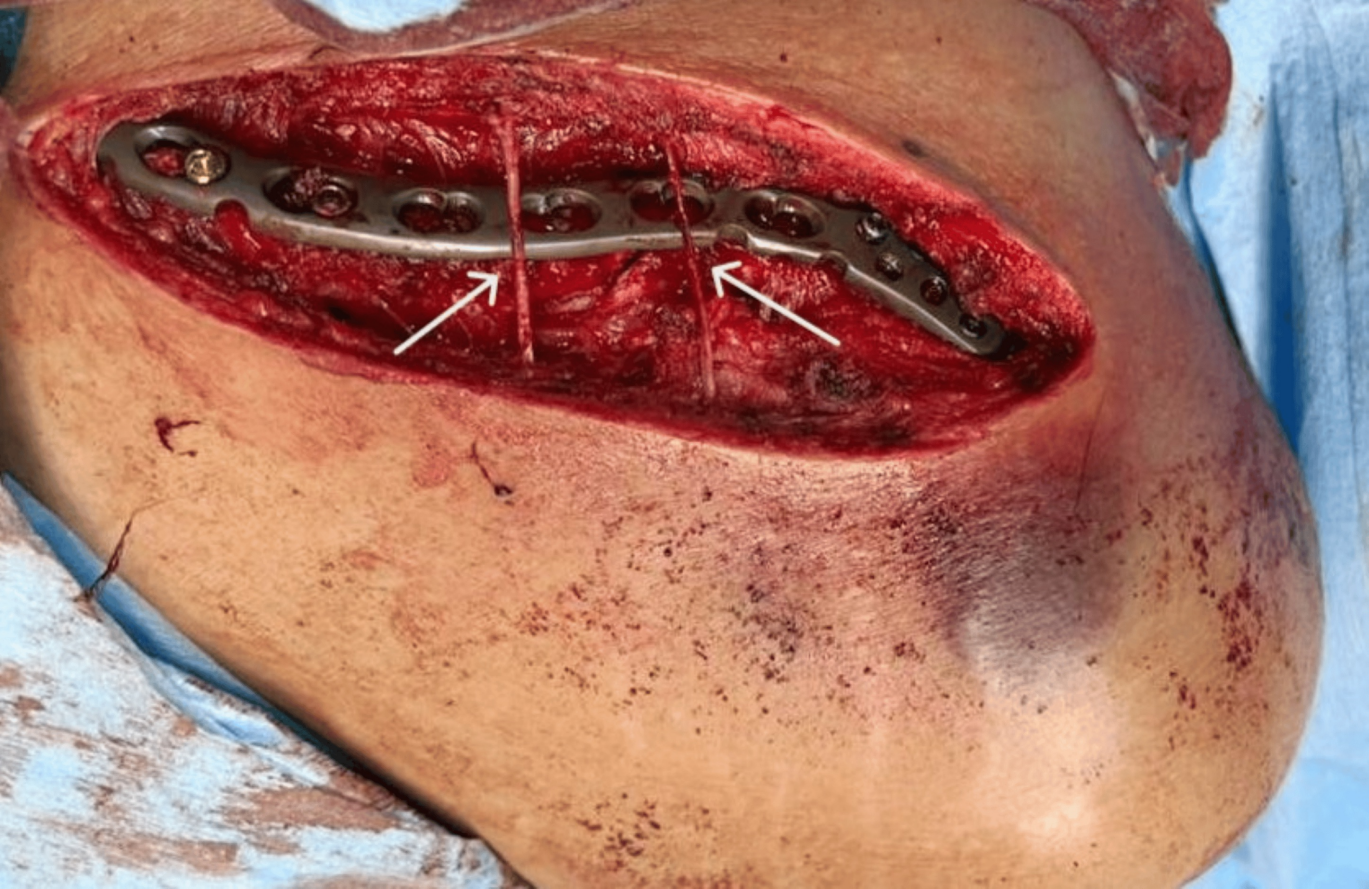
Intraoperative photo The photo depicts fine dissection, preservation of supraclavicular nerves, and fixation using 3.5-mm precontoured LCP LCP: locking compression plate

A sabre-shaped incision was made over the clavicle. The skin and subcutaneous tissue were mobilized as a single layer preserving the supraclavicular nerves (Figure 1). Further, the myofascia over the clavicle was incised as a single layer. The soft tissue was dissected carefully to expose the fracture site. The fracture was fixed with an appropriately sized 3.5-mm precontoured locking compression plate (LCP) for the clavicle, which was applied superiorly. Closure of subcutaneous tissue was done with 2-0 polygalactin suture and skin staples, followed by antiseptic dressing and cuff and collar sling.

Postoperative protocol

The limb of the patient was supported by a cuff and collar sling and the patient was cautioned against any weight lifting. In all cases, range of motion (ROM) exercises were initiated on the second postoperative day. The patient was instructed to remove the cuff and collar sling to do ROM exercises of the elbow and perform pendulum exercises of the shoulder. The patient was taken up for radiological assessment immediately after surgery to assess the fixation and subsequently for follow-up at monthly intervals for at least six months or till the achievement of bony union, whichever was later.

Postoperative assessment and outcome measures

The assessment of final outcomes, both radiological and functional, was done at the end of six months.

Primary Outcome (Radiological)

A standard anteroposterior (AP) view of the clavicle was obtained at each follow-up visit. Radiological features of the union were looked for, such as the gradual disappearance of the fracture line and cortical continuity. The time for the radiological union was noted accordingly.

Primary Outcome (Functional)

The following parameters were assessed with Constant-Murley Score (CMS): postoperative pain, ability to perform activities of daily living, ROM of the shoulder, and power of the shoulder.

Secondary Outcome (Functional)

Complications such as nonunion, malunion, hardware prominence, soft tissue irritation, superficial skin infection, and cosmesis were assessed along with the incidence of implant removal and its causes. The number of cases with complications was expressed as a percentage of the total number of cases available for follow-up.

Statistical analysis

All the measurements and data were analyzed using standard statistical tools. The observations were entered in the Microsoft Excel spreadsheet Version 2021. Normally distributed variables were expressed as means. Analysis was done using the Student's t-test by SPSS Statistics v26 software (IBM Corp., Armonk, NY). A p-value <0.01 was considered statistically significant.

## Results

This prospective observational study involved 26 subjects with displaced clavicular fractures, of them one had a bilateral clavicle fracture. The majority of the patients were in the younger age group, the mean age being 36 years (range: 18-62 years). There was a significant male predominance (77%). Most patients sustained clavicular fractures due to road traffic accidents, of which around 80% were due to two-wheeler accidents, while the rest resulted from car crashes. There was a significant incidence of smoking in the present study with as many as 58% of the patients being smokers. As per the radiological classification of clavicle fractures by Robinson [9], the majority of the cases belonged to Type 2B1. Of the remaining patients, a nearly equal frequency of types 2B2 and 3B1 was observed in the present study. The details related to complications are shown in Table 1.

**Table 1:** Incidence of various postoperative complications

Complications	Frequency	Percentage
Nil	23	85
Implant failure	2	7
Implant irritation	1	4
Prominent postoperative scar/scar irritation	1	4

No complication was observed in 85% of the cases. None of our patients had a postoperative infection, wound dehiscence, nonunion, or postoperative neurological deficit or numbness. However, implant failure was observed in both fractures in the case of the patient with bilateral clavicle fracture within a month of surgery. Implant irritation without prominence was seen in one patient and another had a prominent postoperative scar. The incidence of various postoperative complications in the present study is shown in Table 1.

Reoperation

A reoperation was performed for one patient with a bilateral clavicle fracture where there was implant failure on both sides. It involved implant removal only as the patient did not consent to repeat fixation. This patient was subsequently lost to follow-up.

Time for radiographic union

All the remaining 25 patients achieved radiological union in a mean time of 13.8 weeks with union time ranging from three to five months. The distribution of union time as observed in the present study is presented in Table 2. It was observed that smokers had a longer mean healing time (110 days) compared to non-smokers (83 days). This result was found to be statistically significant (p<0.01)

**Table 2:** Duration for radiographic union

Time for radiographic union	Frequency	Percentage
0-2 months (≤60 days)	0	0
2-3 months (61-90 days)	11	44
3-4 months (91-120 days)	11	44
4-5 months (121-150 days)	3	12
5-6 months (151-180 days)	0	0
Nonunion	0	0

Functional outcome

At the final follow-up at the end of 24 weeks, 25 patients available for follow-up achieved excellent Constant-Murley scores. The mean CMS was 94.9. The functional outcome was excellent in all cases at the final follow-up. The functional outcome of one patient with bilateral clavicle fracture could not be ascertained as he was lost to follow-up after implant removal at two weeks postoperatively and hence could not be included in the final evaluation.

## Discussion

Traditionally, all clavicular fractures were managed conservatively, with open reduction and internal fixation reserved only for symptomatic nonunions in adults because they were commonly seen in the pediatric age group, where healing is not an issue [10-12]. However, with road traffic accidents emerging as the leading cause of all fractures, the incidence of clavicular fractures in adults has risen, which have entirely different characteristics in terms of healing potential, mostly on account of significant displacement, comminution, and systemic/environmental factors like smoking [13,14].

The most significant impasse in the current literature regarding plate fixation is the variations in functional outcome, optimal plate location, and complications like wound dehiscence, nonunion, implant irritation, implant failure, and cosmesis [8,15]. In our series of 26 patients, we analyzed the outcome of managing displaced clavicular fractures with 3.5 mm precontoured LCPs. While the studies by Robinson et al., Sawalha et al., Clement et al., and Napora et al. had a lower percentage of smokers, more than half of the subjects in our study were smokers [8,16,17,18]. This incidental observation indicated the prevalence of this harmful habit on a large scale at least in our region. The mean time for the radiological union in our cohort was 13.8 weeks, slightly more than what was observed in several studies in the literature [19-21]. This could be due to the high prevalence (58%) of smokers in the present study group. It was observed that smokers had a longer mean healing time (110 days) than non-smokers (83 days), which was found to be statistically significant (p<0.01).

Plate irritation was seen in a young lean female patient in our study, probably due to her poor body build. Naimark et al. observed lower implant removal rates with precontoured plates which the authors attributed to lower hardware irritation [22]. In a multicentre study by the Canadian Orthopaedic Trauma Society, no hardware irritation was observed in fractures fixed with precontoured plates; however, plate irritation was common in those managed with limited contact dynamic compression plates/3.5-mm reconstruction plates [3]. Singh et al. reported complications in nine out of 20 cases due to plate irritation, periprosthetic fracture, and infection following fixation with reconstruction plates [23]. Further, Hulsman et al. reported more irritation with IM nails than plates and hence a higher reoperation rate with nails. An anterosuperior approach for using low-profile precontoured LCPs was employed in a study where implant irritation was much lower than nails [24]. A prominent postoperative scar is a complication of plating clavicle fracture and was observed in one female in our study. Similar results have been reported by Hathiwale et al. [25]. 

In our study, implant failure was seen in one patient with a bilateral clavicle fracture, a week after surgery. The patient had been operated on, a few days earlier, for a shaft of femur fracture and subsequently for bilateral clavicle. He started using a walking aid and weight was borne by both upper limbs, prematurely. This led to implant failure and implant removal was done on both sides two weeks postoperatively. He was advised revision fixation, which he refused and was thereafter lost to follow-up. At the final follow-up after six months, no other patient underwent implant removal. This can be attributed to the use of precontoured plates which did not fail if a proper rehabilitation protocol was followed. Postoperative numbness was not observed in our study but was reported in 20% by Mittal et al., which was explained by inadvertent injury to supraclavicular nerves during dissection [26]. The nerves are extremely fine and difficult to identify if the soft tissue dissection is not carefully done. Also, they run perpendicular to the line of incision, and hence a deeper-than-usual incision can lead to inadvertent injury.

Infections reported in studies by Robinson et al., Sawalha et al., Samaiya et al. and Singh et al. were mostly superficial and resolved with a course of antibiotics and aseptic dressing [8,16,21,23]. Sawalha et al. also reported deep infection in one case for which debridement with plate removal was done without further refixation [8]. No case of postoperative infection was observed in the present study. Furthermore, the subcutaneous location and complex architecture of the clavicle make osteosynthesis with standard plates technically challenging. Thus, all patients in this study were operated on using LCPs constituting low-profile 3.5-mm precontoured clavicular plates and lateral extension plates were used when ligamentous instability was suspected in the lateral end. 

Though routine plate removal is not indicated after the fixation of clavicle fractures, problems like plate irritation, implant failure, and infection may warrant plate removal. In the past, plate irritation was reported to be the major cause of implant removal as low-profile plates were not popular. However, with the advent of anatomically contoured low-profile plates, the incidence of plate irritation has decreased. Singh et al. reported reoperations in 43% of cases due to plate irritation and postoperative pain [23]. This could be justified by the fact that the follow-up time was three years. This highlights the importance of using a low-profile plate over the clavicle and these patients should be followed up for a longer time to determine the long-term effects of this surgery.

In the present study, only one patient with a bilateral fracture required revision surgery in the form of implant removal due to early implant failure. This was due to non-compliance with the postoperative rehabilitation protocol. While one patient had plate irritation, it was not significant enough to warrant plate removal. However, a higher incidence of implant removal over a longer follow-up cannot be ruled out on account of implant irritation as the plate is located subcutaneously. In our study, we employed CMS as the measure of the functional outcome as it is easy to carry out and is based on both objective and subjective parameters [27]. The functional outcome in all 25 cases was observed to be excellent (a mean CMS of 94) after the final follow-up at the end of six months, highlighting the importance of proper physiotherapy postoperatively.

One patient had implant failure within a week of fixation in a bilateral clavicle fracture for which he underwent implant removal on both sides. However, the patient refused refixation of the fracture and was lost to follow-up thereafter. The functional outcome in the present study aligns with most studies in the literature that report favorable outcomes after plating clavicle fractures [28]. This could be due to the early and easy postoperative rehabilitation exercises. Further, the functional outcome was reported to be better in non-smokers in the literature. However, more studies with higher sample sizes are required to establish a definite association of smoking with the functional and radiological outcomes of clavicular fractures. In this study, no iatrogenic neurovascular incidents (brachial plexus injury, subclavian artery/vein injury, thoracic outlet syndrome), which are known to be potentially devastating complications, occurred [29]. A depth-stop device to prevent overshooting during drilling along with optimum screw size are prerequisites to prevent such complications. Young surgeons should be wary of these events which, although rare, can be extremely risky.

Limitations

The sample size of the study was relatively small; hence larger studies with longer follow-ups are desirable to validate our findings regarding functional outcomes and long-term complications of using precontoured clavicular plates.

## Conclusions

Based on our findings, using 3.5-mm low-profile precontoured clavicular LCPs is a useful technique that can lead to good outcomes in displaced clavicular fractures to facilitate early union and return of complete function. Good reduction along with preservation of biology are critical to achieve this. Furthermore, smoking was found to significantly delay the mean healing time. Strict abstinence from smoking in any form can speed up the recovery time. Complications in the form of implant irritation and prominent postoperative scar can be avoided by meticulous surgical technique, though such issues should be thoroughly discussed with the patient before performing the surgery.

The rehabilitation process is also of prime significance in enhancing recovery. While the early institution of shoulder exercises is vital, overloading the affected limb in the immediate postoperative period can lead to disastrous results in the form of implant failure, as observed in one of our cases. Thus, caution must be exercised in cases of clavicle fractures associated with lower limb fractures. An early weight-bearing protocol for the lower limb can serve as a risk factor for early implant failure in clavicular fractures managed with plates. Hence, the timing of such surgeries along with their postoperative rehabilitation protocol must be thoroughly planned out before undertaking them.
